# Large-fiber neuropathy in Parkinson’s disease: a narrative review

**DOI:** 10.1186/s42466-024-00354-z

**Published:** 2024-10-28

**Authors:** Eun Hae Kwon, Julia Steininger, Raphael Scherbaum, Ralf Gold, Kalliopi Pitarokoili, Lars Tönges

**Affiliations:** 1 Department of Neurology, St. Josef-Hospital, Ruhr-University Bochum, Bochum, Germany; 2 Neurodegeneration Research, Centre for Protein Diagnostics (ProDi), Ruhr-University, Bochum, Germany

**Keywords:** Parkinson’s disease, Polyneuropathy, Large fiber neuropathy, Etiology, Diagnostics, Management

## Abstract

**Background:**

Numerous studies reported a higher prevalence of polyneuropathy (PNP) in patients with Parkinson’s disease (PD) compared to the general population. Importantly, PNP symptoms can aggravate both motor and sensory disturbances in PD patients and negatively impact the disease course. Recent analyses indicate distinct PNP patterns in PD.

**Main text:**

This review aims to provide an overview of the current insights into etiological factors, diagnostic methods, and management strategies of large fiber neuropathy in PD. Despite the higher prevalence, the causes of PNP in PD are still not fully understood. A genetic predisposition can underlie PNP onset in PD. Main research attention is focused on long-term levodopa exposure which is suggested to increase PNP risk by depletion of methylation cofactors such as vitamin B12 and accumulation of homocysteine that altogether can alter peripheral nerve homeostasis. Beyond a potential “iatrogenic” cause, alpha-synuclein deposition has been detected in sural nerve fibers that could contribute to peripheral neuronal degeneration as part of the systemic manifestation of PD. Whereas mild axonal sensory PNP predominates in PD, a considerable proportion of patients also show motor and upper limb nerve involvement. Intriguingly, a correlation between PNP severity and PD severity has been demonstrated. Therefore, PNP screening involving clinical and instrument-based assessments should be implemented in the clinical routine for early detection and monitoring. Given the etiological uncertainty, therapeutic or preventive options remain limited. Vitamin supplementation and use of catechol-O-methyltransferase-inhibitors can be taken into consideration.

**Conclusion:**

PNP is increasingly recognized as a complicating comorbidity of PD patients. Long-term, large-scale prospective studies are required to elucidate the causative factors for the development and progression of PD-associated PNP to optimize treatment approaches. The overall systemic role of “idiopathic” PNP in PD and a putative association with the progression of neurodegeneration should also be investigated further.

## Background

Parkinson’s disease (PD) is currently one of the fastest-growing neurological conditions worldwide [1, 2]. Deposition of α-synuclein is closely associated with neurodegeneration, which is not limited to striatal dopaminergic and other central nervous regions but extends to peripheral organs and the peripheral nervous system [3]. Based on its multisystemic nature, PD manifests with a broad spectrum of motor and non-motor symptoms. Polyneuropathy (PNP) represents a peripheral nerve dysfunction involving sensory, motor, and autonomic domains [4]. According to the affected nerve structure and type of fibers, PNP can be categorized into axonal versus demyelinating subtype and small (unmyelinated Aδ and C fibers) versus large fiber neuropathy (myelinated Aα and Aβ fibers). For this review, we focus on large fiber neuropathy when referring to PNP.

The first association between PD and PNP was observed in 1991 by a pharmacological study of oral levodopa where PNP was reported as an adverse event [5]. Subsequent studies revealed a higher coincidence of PNP in PD patients, mostly as axonal, predominantly sensory PNP. PNP prevalence varies greatly between studies, ranging from 4.8 to 62% [6–8], whereas PNP rate in the general population of similar age is estimated to be approximately 8% [4, 6]. These divergences in PNP rates could be due to differences in the study population (age, disease duration, therapeutic regimen) and applied diagnostic PNP criteria [9]. PNP symptoms include disturbed sensations such as numbness, paresthesia and pain, sensory ataxia, and muscle weakness and can partially overlap with PD symptoms. For example, pain is an unspecific, yet frequent symptom of PD patients among which neuropathic characteristics can indicate a central, but also a peripheral origin, with a negative impact on the quality of life [10]. Moreover, balance and gait difficulties in PD patients can be aggravated by PNP comorbidity leading to an increased risk for falls [11, 12]. Therefore, this review aims to comprehensively summarize the current views on etiological factors, diagnostics, and treatment options of large fiber neuropathy in PD. For this purpose, a selective literature search was performed using PubMed based on the main search terms “Parkinson’s” in combination with “polyneuropathy”, “peripheral neuropathy” or “large fiber neuropathy”.

## Neuropathology

While large-fiber neuropathy has been more attributed to extrinsic factors, small-fiber neuropathy is considered an intrinsic feature of PD as evidenced by cutaneous denervation and α-synuclein deposits. Doppler et al. reported depositions of phosphorylated α-synuclein in somatosensory and autonomic nerve fibers in skin biopsies of PD patients accompanied by a significant reduction of intraepidermal small nerve fibers [13]. Intraepidermal small nerve fiber density was found to be reduced in untreated as well as levodopa-treated PD patients compared to controls [14]. In contrast, Podgorny et al. did not observe any difference in epidermal nerve densities between early untreated PD patients and controls [15]. Instead, small-fiber neuropathy in PD was demonstrated by reduced corneal nerve fiber densities suggesting corneal confocal microscopy as a more sensitive tool for early detection of preclinical small fiber loss. In a longitudinal study, a higher oligomeric α-synuclein load was detected in skin biopsies of patients with PD and multiple system atrophy compared to controls and tauopathies, yielding a high diagnostic performance for synucleinopathies [16]. Interestingly, PD patients displayed a small nerve fiber pathology with progressive denervation after two years of follow-up, highlighting the potential of small-fiber neuropathy as a progression marker in PD.

Due to their invasiveness, nerve biopsies for evaluation of large-fiber nerve alterations were reserved for individual severe PNP cases under enteral or high-dose oral levodopa therapy. Sural nerve biopsies revealed a severe axonal degeneration with a marked reduction in myelinated nerve fiber density, accompanied by endoneurial edema and myelin debris in endoneurial macrophages [17]. Inflammatory changes with perivascular lymphocytic cuffing have also been described [18]. In the superficial peroneal nerve, intra-axial ubiquitin aggregates were found to be more numerous in PD patients with PNP compared to those without PD as possible correlates of the underlying neurodegenerative process [19]. For the first time, Zhang et al. verified the deposition of phosphorylated α-synuclein in sural nerves in vivo in all 16 PD patients and none of the 15 controls [20]. Expression of α-synuclein was mainly located in Schwann cells, but scarcely in axons. The same study group demonstrated peripheral nerve inflammation by increased expression of glial fibrillary acidic protein and inflammatory cytokines (IL-1ß, IL-6, TNF-a) in activated Schwann cells of PD patients irrespective of the presence of sensory disturbances [21]. Different expression patterns of phosphorylated α-synuclein and tau protein in sural nerve biopsy even allowed differentiation of PD from the atypical Parkinsonian syndromes multiple system atrophy and progressive supranuclear paralysis [22]. In summary, these findings support the hypothesis of a peripheral origin of α-synuclein pathology and the multisystemic nature of PD, yet the pathogenicity of α-synuclein deposition needs to be clarified. Therefore, clinicopathological correlations by means of nerve biopsy are mandatory to validate peripheral alpha-synuclein pathology as a diagnostic biomarker and further elucidate the underlying pathomechanisms of PNP in PD.

## Etiology

Despite the higher PNP rate in PD, the exact linkage between these two disease entities is still unclear. Age represents a risk factor for developing both PD and PNP. Ceravolo et al. calculated an increase of PNP risk in PD by approximately 8% for each year of age [6]. Age-dependent nutritional deficiencies such as decreased vitamin B12 levels have been discussed to partly influence the onset of PNP in PD [23]. Yet, the PNP rate in PD is higher than in age-matched controls indicating a higher susceptibility of the PD population. The leading causes of PNP are diabetes, followed by alcohol abuse, toxic agents, vitamin deficiencies, immune-mediated causes, and hereditary factors, whereas 20–30% of PNP cases remain idiopathic [24]. Toth et al. found that 30% of PD patients with PNP had a defined cause for PNP including diabetes, monoclonal gammopathy, and chronic inflammatory demyelinating peripheral neuropathy [25]. The PD population shows a range of comorbidities including cardiovascular diseases and diabetes [26, 27]. Interestingly, diabetes has been associated with increased PD risk [28]. Evidence supports the idea of shared pathomechanisms allowing the repurposing of antidiabetic agents like glucagon-like peptide-1 receptor agonists for neuroprotective effects in PD. Focusing on truly idiopathic PNP will allow a better understanding of the relationship between PD and PNP. However, PNP-causing comorbidities should be taken into consideration to provide a more holistic estimation of PNP prevalence in PD.

### Genetics

The occurrence of PNP has been reported in hereditary forms of parkinsonism [29]. A nerve conduction study revealed that 8 of 9 patients with juvenile parkinsonism with PARK2-mutation showed reduced sural nerve amplitudes [30]. PARK2 gene encodes an E3 ubiquitin ligase termed Parkin which plays an important role in proteasomal degradation. Interestingly, PARK2 is expressed in sural nerves [31]. It is hypothesized that proteasomal impairment caused by loss of Parkin function could contribute to neurotoxicity, although the effects on peripheral nerves remain unknown. Another candidate gene to increase susceptibility for PNP could be the MTHFR gene encoding the methylenetetrahydrofolate reductase (MTHFR), an essential enzyme for homocysteine metabolism [32]. Mutation of the MTHFR gene could lead to an elevation of homocysteine levels which is associated with an increased risk for PNP due to its peripheral toxic effects [33]. For PD patients, this mutation is particularly critical since it can aggravate levodopa-induced hyperhomocysteinemia. Moreover, an association of MTHFR polymorphisms with increased risk for developing PD is under debate.

### Levodopa metabolism and vitamin deficits

In their pioneering study, Toth et al. demonstrated a significant association between cumulative lifetime levodopa dosage and fasting methylmalonic acid (MMA) levels with PNP severity [25]. Since then, further studies supported the hypothesis that levodopa may play a causative role in the development of PNP in PD, possibly related to its metabolic products [6, 32, 34, 35].

Levodopa’s metabolic cycle starts with methylation by catechol-O-methyltransferase (COMT) using S-adenosylmethionine (SAM) as a methyl donor [32]. Demethylation of SAM produces S-adenosylhomocysteine (SAH) which is hydrolyzed to homocysteine. Homocysteine enters either the remethylation pathway requiring vitamin B12 and folate cofactors or the trans-sulfuration pathway for the production of cysteine involving vitamin B6 as a cofactor. The latter pathway leads to the formation of L-methylmalonyl-coenzyme A which in case of vitamin B12 deficiency results in the accumulation of MMA, an indirect marker of vitamin B12 deficiency. Consequently, chronic levodopa intake can lead to the accumulation of homocysteine and MMA as well as depletion of vitamin B6, B12, and folate, each of which can alter peripheral nerve homeostasis. Homocysteine can exert neurotoxicity by overstimulating glutamate receptors, promoting oxidative stress and DNA hypomethylation, and is linked to various neurological and cardiovascular conditions [36]. Vitamin B6 plays a key role in the synthesis of neurotransmitters, whereas vitamin B12 is particularly involved in nerve regeneration and remyelination [37].

In a large multicenter study with 330 PD patients and 137 controls, PNP risk was stratified according to levodopa exposure [6]. Overall, 19.40% of patients with ≥ 3 years of levodopa exposure, 6.80% with < 3 years of levodopa exposure, 4.82% in the dopa-naïve group, and 8.76% in the control group were diagnosed with PNP-identifying levodopa as a main risk factor. The risk of PNP was 2.38-fold higher in the long-term treated group compared to the control group. This finding is supported by a study of a Romanian cohort reporting a significantly higher PNP prevalence in PD subjects on levodopa treatment compared to untreated PD subjects [38]. In this study, lower vitamin B12 levels correlated with higher levodopa daily dose and treatment, levodopa dose correlated inversely with the nerve amplitudes. In contrast, Shahrizaila et al. observed no difference in the PNP rate between levodopa-naïve and levodopa-treated PD patients [39]. Another study confirmed a higher PNP prevalence in the treated group compared to the untreated group, though concluded after regression analysis, that the levodopa effect was only contributory and surpassed by age and folate levels [23]. Higher homocysteine levels have been reported in PD patients with PNP correlating with levodopa intake [40], but also in drug-naïve de novo PD patients suggesting that homocysteine is independently elevated in PD [41]. A study of an Indian cohort found no difference in homocysteine, vitamin B12, and folate levels between PD patients and controls, however, the study population exhibited a low overall PNP rate of 9.68% [42].

The impact of levodopa therapy on PNP risk has been shown to be more pronounced with levodopa/carbidopa intestinal gel (LCIG) infusion. A systematic review reported a higher occurrence of PNP under LCIG (42.1%) in comparison to oral levodopa treatment (30.2%) [32]. PNP characteristics differed between the different administration modes as LCIG-treated subjects displayed a sensorimotor PNP with sub-/acute onset, rarely with demyelinating features, compared to the more commonly observed mild chronic axonal predominantly sensory PNP in levodopa-treated patients. In a prospective study of 23 PD patients, two patients developed a subacute PNP, two patients a chronic PNP, and seven patients a subclinical PNP two years after starting LCIG therapy [43]. Mancini et al. studied PNP characteristics under different therapeutic regimens including LCIG, oral levodopa, and other dopaminergic medications [44]. PNP prevalence was shown to be increased under levodopa therapy, independent of the route of administration. The majority of cases exhibited a subacute sensory PNP, whereas 29% of LCIG patients manifested an acute demyelinating motor form. Vitamin B12 and folate levels were significantly lower, homocysteine levels were significantly higher in levodopa-treated compared with non-levodopa-treated patients. Loens et al. found that only vitamin B6 and homocysteine levels correlated with levodopa dose and concluded that PNP risk increased with higher levodopa dose accumulated by longer disease duration and greater bioavailability due to intestinal administration [45]. Furthermore, PNP has been associated with weight loss, which is a frequent side effect of LCIG therapy [46]. It has been suggested that the viscous enteral gel might hamper intestinal membrane function leading to malabsorption that could result in vitamin B and folate deficiencies further exacerbating PNP severity [17, 47]. Recently, continuous subcutaneous foslevodopa/foscarbidopa infusion has been introduced as an additional treatment option for advanced PD. So far, over the short term of 12 weeks, the incidence of weight loss and PNP was found to be equally low between oral and subcutaneous levodopa application forms (1% weight loss, 3% PNP in both groups) [48]. Only long-term data will answer the question of whether subcutaneous application differs from intestinal application of levodopa in terms of PNP risk. Taken together, these findings point towards a strong relationship between chronic levodopa intake and PNP risk, although the exact causal pathomechanisms and associations with one-carbon metabolites need to be further elaborated. Publications addressing PNP prevalence and associated findings in PD are listed in Table 1.

**Table 1:** Overview of PNP status in PD

Authors	Medication	Samples	Examined nerves	PNP prevalence	Associated findings
Toth et al. [25]	Oral LD	500 PD	Sural, peroneal, tibial	9.8% of PD patients	Hyc/MMA↑ Cumulative LD dose and MMA level were associated with PNP severity
Toth et al. [34]	Oral LD	58 PD, 58 controls	Peroneal, tibial, median, ulnar	55% of PD patients vs. 9% of controls	Age↑, UPDRS scores↑, Hyc/MMA ↑ PNP severity correlated with LD exposure and MMA level
Ceravalo et al. [6]	Oral LD	330 PD, 137 controls	Sural, peroneal	19.40% of PD patients with long LD exposure, 6.80% with short LD exposure, 4.82% with no LD exposure vs. 8.76% of controls	LD dose↑, Hyc↑, vitamin B12↓
Shahrizaila et al. [39]	Oral LD vs LD-naïve	51 PD	Sural, tibial, median, ulnar	23% of LD-treated vs. 24% of LD-naïve PD patients ( ↔)	MMA/Hyc ↔
De Araújo et al. [49]	Oral LD	38 PD, 16 PS	Peroneal, tibial, sural	34.2% of PD patients vs. 23.5% of patients with parkinsonism other than PD ( ↔)	EMG abnormalities correlated with age and Hoehn & Yahr stage
Vanta et al. [38]	Oral LD vs LD-naïve	73 PD	sural, peroneal, tibial	67.3% of LD-treated vs. 4.8% of LD-naïve PD patients	Vitamin B12 level correlated with LD daily dose and duration of treatment, LD dose correlated with sural nerve amplitude
Lee and Baik [41]	LD-naïve	105 de novo PD	Median, ulnar, sural, peroneal	22.8% of PD patients PD	Hyc↑
Kühn et al. [7]	Oral LD	50 PD	Tibial, median, sural	62% of PD patients	Motor nerve amplitudes correlate with Hoehn & Yahr stage and UPDRS III score
Mathukumalli et al. [42]	Oral LD	93 PD, 70 controls	median, ulnar, peroneal, tibial, sural	7.53% of PD patients vs. 4.29% in controls ( ↔)	Vitamin B12/Hyc ↔ between cases and controls
Ramachandran et al. [35]	Oral LD	154 PD	median, ulnar, peroneal, tibial, sural	18.2% of PD patients	Disease duration and severity, cumulative LD dose and hcy level were associated with PNP
Corrà et al. [12]	Oral LD	99 PD	Sural, tibial, peroneal, ulnar, radial, median	12.5% of PD patients	Presence of PNP was associated with gait and balance parameters
Jugel et al. [46]	Oral PD medication vs. LCIG	30 PD	median, tibial, sural, peroneal	66.7% of PD patients with oral PD medication vs. 100% LCIG-treated patients	Degree of neuropathic change correlated with weight loss and LD dose in LCIG group
Mancini et al. [44]	Oral LD vs. LCIG vs. non-LD	150 PD	ulnar, peroneal, tibial, sural	28% of LCIG-treated patients vs. 20% with oral LD vs. 6% with non-LD treatment	Vitamin 12↓, folate↓, hcy↑ in LD-treated patients. LD daily dose correlated with Hcy levels
Loens et al. [45]	Oral LD vs. LCIG	21 PD	median, peroneal, tibial, sural	100% of LCIG-treated patients vs. 72.7% with oral LD	Vitamin B12, folate, hyc ↔ LD dose correlated with vitamin B6 deficiency
Kwon et al. [50]	Oral LD	41 PD	tibial, sural, peroneal, median, ulnar, radial	65.85% of PD patients	PNP deteriorated in 21.95% of cases. Median sensory nerve was most affected. Sural nerve amplitude correlated with lower quality of life and worse cognition

*LD*, levodopa; *LCIG*, levodopa/carbidopa intestinal gel; *PS*, parkinsonism; *Hyc*, homocysteine; *MMA*, methylmalonic acid; *UPDRS*, Unified Parkinson’s Disease Rating Scale; ↑ elevated; ↓reduced; ↔ not significantly different

## Diagnostics

According to the American Academy of Neurology (AAN) recommendations, the most accurate diagnosis of distal PNP is obtained from the combination of clinical symptoms, signs, and electrodiagnostic findings [51]. Signs are considered to better predict PNP than symptoms, whereas electrodiagnostic studies would provide a higher level of specificity to PNP diagnosis. Nerve conduction studies in the PD population have used different diagnostic approaches to define PNP. For example, some studies applied the recommended PNP criteria by AAN [6, 34, 42], and other studies solely relied on the electrodiagnostic findings for PNP diagnosis causing a great variability of PNP rates [7, 12, 41]. For better comparability among studies, a standardized implementation of the AAN PNP criteria would be preferable. Yet, electrodiagnostic studies can capture early subclinical nerve alterations particularly useful for longitudinal evaluation of PNP progression that is required to better understand PNP involvement in PD.

### Clinical scores

Since PD-specific rating scales only cover a few aspects of neuropathic manifestations and are rather unspecific, common clinical scores used for the evaluation of diabetic neuropathy can be applied for PNP screening in PD patients focusing on large fiber neuropathy [52]. In this review, relevant PNP scoring systems that have been utilized for the investigation of PD-related PNP are introduced. The Neuropathy Symptom Score (NSS) and the Neuropathy Disability Score (NDS) were originally proposed by Dyck et al. to assess the quality and severity of neuropathic symptoms and neurological deficits [53]. These scoring systems have been modified over time to facilitate clinical application [54]. The NSS is a patient questionnaire categorized in symptomatology, localization, exacerbating, and improving circumstances. Symptom severity is classified into mild (3–4 points), moderate (5–6 points) and severe neuropathy (7–10 points). Kühn et al. reported that 86% of PD patients experienced at least one neuropathic symptom in the NSS [7]. NSS also correlated with motor (UPDRS part II and III) and non-motor (NMS-Quest, PDQ-39) PD scores. The NDS is based on the clinical examination of neuropathic signs on both sides including ankle jerk reflex, vibration, pinprick, and temperature sensation. The maximum total score is ten points. The clinical scores NSS and NDS were found to correlate with electrophysiological data in diabetic neuropathy [55]. The Toronto Clinical Scoring System (TCSS) is a neuropathy assessment instrument combining the scoring of all three domains of neuropathic symptoms, reflex status, and sensory testing, with a maximum score of 19 points [56]. Toth et al. used TCSS to assess PNP severity and found a correlation between PNP severity and PD severity as reflected by the Unified Parkinson’s Disease Rating Scale (UPDRS) scores [34].

### Electrophysiological diagnostics

Nerve conduction studies (NCS) are a reliable and objective method to evaluate large nerve fiber dysfunction and are therefore regarded as the gold standard for diagnosing and monitoring PNP [57]. Axonal nerve damage, most common for PD-related PNP, is detected by reduced compound muscle action potential (cMAP) in motor nerves or reduced sensory nerve action potential (sNAP). Demyelinating neuropathies have been reported in rare cases of PD patients with acute PNP onset and present with reduced conduction velocities and prolonged distal and F-wave latencies. The simplified NCS protocol of the AAN guidelines recommends the unilateral screening of the sural sensory or peroneal motor NCS [51]. In case of an abnormal finding, NCS should be extended to the ulnar (motor, sensory) and median (sensory) nerve. If a response of a studied nerve is absent, the contralateral nerve can be measured. If no peroneal motor response can be obtained, the ipsilateral tibial nerve can be studied. A nerve conduction abnormality (≥ 99th or ≤ 1st percentile) in at least two separate nerves, one of which must be the sural nerve, constitutes the minimal criterion for the electrophysiological diagnosis of a PNP. However, studies deviated from these recommendations since this protocol is time-consuming and requires special expertise leading to discrepant results. Firstly, the range of the examined nerves varied between studies. Some studies only focused on the nerves of the lower limbs neglecting nerve involvement of the upper limbs [6, 40, 49]. In our recently published longitudinal study, we observed a PNP deterioration in 21.95% of PD cases over two years [50]. The median sensory nerve was most frequently affected with the strongest nerve amplitude reduction of 45.0%. Secondly, deviant cut-off reference values were used to determine abnormal NCS. For example, Kühn et al. referred to sural sNAP < 3.6 mV for elderly patients as abnormal [7], whereas Araújo et al. tolerated absent sural responses in patients older than 60 years resulting in lower PNP rate [49]. Furthermore, Kühn et al. increased sensitivity for early PNP detection by choosing the lower value of bilateral conduction. Thirdly, the grading of PNP severity differed between studies. Kühn et al. categorized into mild sensory (reduced sural sNAP only), moderate sensorimotor (additionally reduced tibial cMAP), and severe sensorimotor PNP (additionally reduced median cMAP) resulting in 14 mild, 11 moderate, and 6 severe PD cases. Another study differentiated between 15 mild sensory (reduced sNAP), 8 severe sensory (absent sNAP), and 5 sensorimotor PNP (reduced sNAP and cMAP) [35]. These findings altogether illustrate the challenges of the inconsistencies of the PNP definition and thus diminished study comparability. An extended electrophysiological status is yet of importance to evaluate PNP severity since previous findings indicate a significant involvement also of motor and upper limb nerve impairment. Several studies have reported an association between the extent of PNP severity and PD severity (UPDRS, Hoehn and Yahr stage) [7, 34, 35]. Kühn et al. found a reduced tibial nerve amplitude in PD patients with higher Hoehn and Yahr stages and compromised UPDRS III score [7]. In the longitudinal analysis, nerve amplitudes correlated with higher motor and also non-motor scores at baseline (PD-related quality of life, cognition scores) [50]. Although no statistically significant correlation could be established between PNP progression and PD progression over the disease course, a significant deterioration of both conditions could be observed. Therefore, large fiber neuropathy can affect PD severity and parallel PD progression as a manifestation of the underlying peripheral neurodegenerative process. Merola et al. suggested PNP as an independent peripheral marker of a severe PD phenotype associated with worse cognitive, axial motor, autonomic, and nonmotor features [58]. Further longitudinal studies of nerve conduction abnormalities are urgently needed to investigate the natural course of PNP in PD. Screening and monitoring of PD patients for PNP comorbidity should be implemented into clinical routine and be particularly considered when PD patients experience clinical worsening. Needle electromyography (EMG) may serve as a supplementary instrument to determine the chronicity of an axonal neuropathic process [57]. The presence of spontaneous muscle fiber activity at rest indicates denervation, whereas reinnervation is assessed by the motor unit action potential on voluntary muscle contraction. Since this technique is laborious and painful for the patient, its indication should be considered carefully only if relevant additional information can be obtained, for example in case of acute PNP onset during LCIG treatment and monitoring such PNP course.

### Peripheral nerve imaging

High-resolution nerve ultrasound (HRUS) is a non-invasive modality to visualize morphologic alterations of peripheral nerves [59]. Kühn et al. applied HRUS for the first time to investigate PD-related PNP [7]. In their study, a higher prevalence of enlarged cross-sectional areas was detected in PD patients with PNP compared to those without PD, mostly at typical entrapment sites without clinical or electrophysiological correlate suggesting an increased nerve vulnerability in PD [7]. Magnetic resonance (MR) neurography is an imaging method to capture the morphological changes and precise location of nerve injury using MR imaging. This method has been already established as a supportive diagnostic tool for chronic inflammatory demyelinating polyradiculoneuropathy [60] but has not been studied for PD-related PNP so far. Allowing a meticulous depiction of nerve alterations, this non-invasive method might be also useful as a tool for evaluating PNP in PD and should be studied in the future.

### Nerve biopsy

A nerve biopsy, typically of the sural nerve, is an invasive procedure that leaves the patients with a sensory deficit. It should be considered as a final step in the diagnostic work-up of neuropathies of unknown origin and if an inflammatory or other potentially treatable etiology is suspected [61]. As previously discussed, for research purposes, nerve biopsies are indispensable since they can provide important in vivo clinicopathological correlations and clarify the relevance of peripheral α-synuclein deposition that truly helps unravel the pathogenic link between PNP and PD.

## Management

### Symptomatic approach

Regarding clinical management, symptomatic and causative therapeutic approaches can be distinguished. PNP can cause neuropathic pain that significantly impacts quality of life and therefore requires symptomatic relief. The Neuropathic Pain Special Interest Group proposed gabapentinoids gabapentin and pregabalin, tricyclic antidepressants (TCAs), and serotonin-norepinephrine reuptake inhibitors (SNRI) venlafaxine and duloxetine as first-line drugs for neuropathic pain irrespective of the cause [62]. Gabapentin was found to have positive effects on PD motor symptoms advocating its use for neuropathic pain with additional motor benefits [63, 64]. Furthermore, duloxetine was tested for treatment of central pain in an open-label trial and 13 of 20 PD patients reported varying degrees of pain relief [65]. Despite their effectiveness, TCAs should be used with caution due to the anticholinergic side effects that can cause sedation, deteriorate cognition, or induce psychosis, particularly in the elderly more advanced PD patients. Topical treatment with lidocaine or capsaicin can be considered a second-line option. In severe cases, strong opioids are recommended as third-line drugs. In a double-blind placebo-controlled phase 2 study, treatment with prolonged-release oxycodone-naloxone showed a positive effect on PD-related severe pain [66]. Gait disturbance and postural instability in PD can be aggravated by comorbidity with PNP. Physical exercise with a special focus on balance and gait training has shown great therapeutic potential to improve proprioception, gait performance, postural control, and prevention of falls in patients with peripheral neuropathy [67], but also in patients with PD [68]. The beneficial effects of exercise intervention for neuropathy were demonstrated by an experimental study of rodents following peripheral nerve injuries, showing increased axon regeneration, muscle reinnervation and increased expression of neurotrophic factors [69]. Therefore, exercise should be implemented as an integral therapeutic part targeting gait and balance disturbances as overlapping symptoms of PD and PNP. The optimal fall prevention protocol for individuals with PD and PNP needs to be determined in further studies.

### Causative approach

Since levodopa-induced depletion of methyl group-donating vitamins and cofactors and accumulation of homocysteine and MMA are implied in the pathogenesis of PD-related PNP, treatment approaches are based on vitamin supplementation and use of COMT-inhibitors. In one study, PD patients with idiopathic PNP and patients with idiopathic PNP only identified with abnormalities in cobalamin, homocysteine, or MMA levels and were treated with monthly intramuscular injection of 1000 µg cobalamin for 1–2 years [25]. In most cases, the abnormal values were normalized upon treatment. Clinical assessment and electrophysiological parameters were stable at follow-up in the PD with PNP subgroup compared to the mild PNP deterioration in the other subgroup. The authors interpreted that one explanation for this finding could be an iatrogenic cause of levodopa-induced vitamin B12 dysmetabolism, alternatively, PNP could represent a peripheral nervous system manifestation of PD. In an open-label study, 30 PD patients were treated on an oral combination supplementation of vitamin B12, folic acid, vitamin B6, and vitamin B2 for 10 days a month since LCIG started and throughout the follow-up [70]. The authors reported a low incidence of PNP (19%) and only mild electrophysiological progression in patients with pre-existing PNP. Ultimately, despite normal vitamin levels and appropriate supplement therapy, the development of PNP cannot be prevented, however, the course of PNP progression could be attenuated.

There are recommendations for PD patients undergoing LCIG therapy [71, 72]. Before starting LCIG, 6 months after, and once a year thereafter, laboratory assessments should be performed including vitamins B12, B6, and folic acid. Since 50% of cobalamin-deficient patients present with normal serum cobalamin, determination of homocysteine and MMA levels is suggested (the latter is more specific as an early marker of functional vitamin B12 deficiency). NCS status should be evaluated at baseline and once yearly. For high-risk patients (pre-existing neuropathy, advanced PD) prophylactic vitamin B12 supplementation can be considered. If laboratory abnormalities or PNP diagnosis are present, supplementation therapy should be initiated. A common regimen for vitamin B12 would start with 1000 μg intramuscularly for 5–7 days followed by a monthly 1000 μg intramuscular injection. Folate can be administered orally at the dosage of 5 mg daily. Vitamin B6 supplementation is not recommended because it interferes with the actions of the decarboxylase inhibitor and has neurotoxic properties in excess levels [32].

**Fig. 1:**
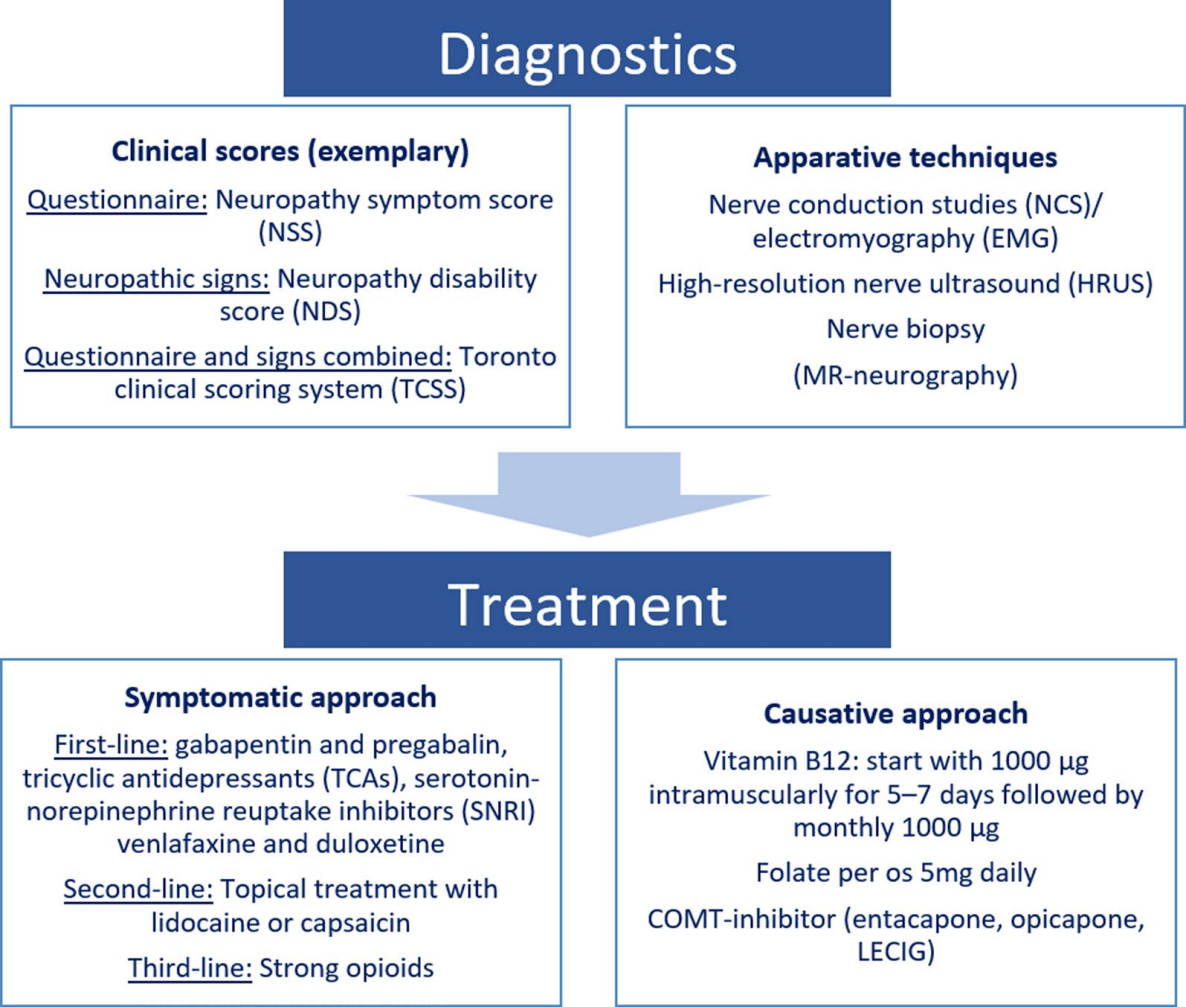
Diagnostic and therapeutic options for PNP in PD

Methylation of levodopa by the enzyme COMT represents a critical initial step for vitamin B12 depletion and homocysteine accumulation. Therefore, COMT inhibition could have a protective effect on restoring these metabolic imbalances and thus preventing the development of PNP in PD patients. In a multicenter study of 197 PD patients, a significantly lower PNP prevalence was observed in PD patients co-administered with the COMT-inhibitor entacapone for at least 18 months (5.7%) compared to those without COMT-inhibitor under levodopa exposure for at least 3 years (19.4%) [73]. Higher serum vitamin B12 levels and lower homocysteine levels were measured in patients on COMT inhibitors. In contrast, a study of a heterogenous PD cohort undergoing different therapeutic regimens did not find a difference in PNP prevalence, vitamin B12, and homocysteine levels in patients on COMT-inhibitor [44]. However, the latter study did not specify the treatment duration of levodopa or COMT-inhibitor, which could be relevant to the possible changes in the PNP status. Higher levodopa bioavailability is achieved by the COMT-inhibitor opicapone compared to entacapone, whereas both compounds could prevent an increase in homocysteine [74]. Levodopa-entacapone-carbidopa intestinal gel (LECIG) is a novel infusion therapy option for advanced PD containing the COMT-inhibitor entacapone in addition to levodopa and carbidopa. Notably, a single-center study of 30 PD patients reported no clinically diagnosed cases of PNP and no increase in homocysteine level during the first 6 months of LECIG treatment [75]. However, the authors conceded that many patients used over-the-counter vitamin supplements, and no routine NCS was performed. Figure 1 summarizes diagnostic and therapeutic options for PNP in PD.

## Conclusions

The higher PNP prevalence in PD indicates a pathophysiological relationship between these distinct conditions probably as a result of a complex interplay between genetic predisposition, extrinsic risk factors influencing peripheral nerve homeostasis, and an intrinsic peripheral neurodegenerative process. Evidence suggests that the cumulative exposure to levodopa is critical for PNP risk. More studies on dopa-naïve PD patients are required to unmask a truly “idiopathic” form of PNP. To further elucidate pathomechanisms, neuropathologic correlations are mandatory despite invasiveness. The pathogenic role of α-synuclein deposits in peripheral nerves needs to be clarified. PNP should be recognized as an important aspect of PD and therefore adequately detected and monitored by comprehensive NCS and clinical scores. Large-scale prospective studies will help understand PNP progression in PD and validate biomarker potential, stratify risk factors for developing PD, and evaluate the protective role of vitamin supplementation and use of COMT inhibitors. The long-term results of the novel PD treatment options such as subcutaneous levodopa infusion therapy and LECIG remain to be seen.

## Data Availability

All data generated or analysed during this study are included in this published article.

## Abbreviations

AAN: American Academy of Neurology
COMT: Catechol-O-methyltransferase
CMAP: Compound muscle action potential
EMG: Electromyography
HRUS: High-resolution ultrasound
LCIG: Levodopa/carbidopa intestinal gel
LECIG: Levodopa-entacapone-carbidopa intestinal gel
MMA: Methylmalonic acid
MR: Magnetic resonance
MTHFR: Methylenetetrahydrofolate reductase
NCS: Nerve conduction studies
NDS: Neuropathy Disability Score
NSS: Neuropathy Symptom Score
PD: Parkinson’s disease
PNP: Polyneuropathy
SAH: S-adenosylhomocysteine
SAM: S-adenosylmethionine
SNAP: Sensory nerve action potential
SNRI: Serotonin-norepinephrine reuptake inhibitor
TCA: Tricyclic antidepressant
TCSS: Toronto Clinical Scoring System
UPDRS: Unified Parkinson’s Disease Rating Scale
